# Nonoperative Management Recommendations for Knee Osteoarthritis: A Review of Clinical Guidelines and Treatment Alternatives

**DOI:** 10.7759/cureus.95540

**Published:** 2025-10-27

**Authors:** Derick Rodríguez-Reyes, Ricardo Vargas-Figueroa, Amanda S Vázquez-Lloret, Hiram E Luigi Martinez, Gabriel Gonzalez-Diaz, Rafael Señeriz Ortiz

**Affiliations:** 1 Department of Orthopaedic Surgery, Ponce Health Sciences University, Ponce, PRI

**Keywords:** exercise, injections, knee osteoarthritis/koa, orthobiologics, osteoarthritis, pharmacologic therapy, weight loss

## Abstract

Knee osteoarthritis is among the leading causes of disability worldwide, imposing a substantial physical, psychological, and economic burden. Nonoperative management remains the cornerstone of initial care for patients before surgical considerations. This narrative review aims to synthesize current international guidelines and supporting evidence for both traditional and emerging nonoperative therapies, providing a pragmatic framework for clinical practice. Evidence consistently supports core interventions, such as patient education, self-management, weight reduction, and structured exercise. Pharmacologic adjuncts, including topical and oral nonsteroidal anti-inflammatory drugs (NSAIDs), may provide symptomatic relief, while intra-articular corticosteroids are appropriate in selected cases. More recent approaches, such as platelet-rich plasma, genicular nerve radiofrequency ablation, and genicular artery embolization, demonstrate early promise, though long-term comparative data remain limited. Optimal outcomes are achieved through patient-centered, multidisciplinary care models that address barriers, like poor adherence to exercise and limited access to weight-loss programs. This review was conducted via targeted PubMed searches using terms related to “knee osteoarthritis,” “non-operative treatment,” “guidelines,” “exercise,” “weight loss,” “NSAIDs,” “intra-articular corticosteroids,” “emerging therapies,” “hyaluronic acid,” “platelet-rich plasma,” “radiofrequency ablation,” and “genicular artery embolization.” Our synthesis followed the Scale for the Assessment of Narrative Review Articles (SANRA) recommendations for narrative reviews to ensure methodological rigor. Effective nonoperative care depends on implementing guideline-concordant strategies, recognizing emerging modalities, and tailoring therapy to patient phenotype. Future priorities include scalable models of care, high-quality comparative effectiveness trials, and closing the gap between guideline recommendations and real-world practice to improve pain, function, and quality of life.

## Introduction and background

Knee osteoarthritis (OA) is one of the most common causes of chronic pain and disability worldwide, creating substantial physical, psychological, and economic burden for both patients and healthcare systems [1-3]. The clinical impact is considerable: up to 80% of affected individuals experience mobility limitations, and approximately one quarter struggle with basic activities of daily living [1,2]. The reported chronic pain is often accompanied by deconditioning, mood disturbance, and increased healthcare utilization, further compounding its societal costs [2,3].

Because definitive management relies on operative intervention, nonoperative alternatives remain focused on symptom control and functional improvement [1,3]. Current clinical practice guidelines from the American Academy of Orthopaedic Surgeons (AAOS) recommend a multimodal approach, with particular emphasis on exercise and weight loss, especially in individuals with obesity [4]. Exercise has consistently shown modest but sustained improvements in pain and function, while weight reduction may provide additive benefit when combined with exercise [2].

Adjunctive options include self-management strategies and education programs, which demonstrate measurable improvements in pain and physical function [1]. Pharmacologic treatments, particularly topical and oral nonsteroidal anti-inflammatory drugs (NSAIDs), offer symptom relief when lifestyle measures are insufficient [2]. And in cases with limited benefit from lifestyle modifications and oral medications, intra-articular corticosteroid injections may be considered for short-term management of refractory pain, although their overall benefit remains limited [1,3].

Taken together, nonoperative strategies represent the cornerstone of care in knee OA, with the ultimate goals of reducing pain, improving function, and maintaining quality of life. This review summarizes current guideline-based recommendations and recent evidence supporting both traditional and emerging approaches to nonoperative management.

## Review

Methodology

This narrative review was conducted in accordance with the Scale for the Assessment of Narrative Review Articles (SANRA) [5] recommendations to ensure methodological rigor and transparency in narrative synthesis. While we incorporated elements of structured searching, this work was not designed as a systematic review and did not include a preregistered protocol, formal risk of bias assessment, or meta-analysis.

A literature search was performed in PubMed/MEDLINE up to August 2025 using combinations of key terms including “knee osteoarthritis,” “non-operative treatment,” “guidelines,” “exercise,” “weight loss,” “NSAIDs,” “intra-articular corticosteroids,” “emerging therapies,” “hyaluronic acid,” “platelet-rich plasma,” “radiofrequency ablation,” and “genicular artery embolization.” Selection emphasized international guidelines, randomized controlled trials, meta-analyses, and high-quality observational studies that inform contemporary practice.

Studies addressing non-operative management of knee OA, including non-pharmacologic, pharmacologic, and procedural interventions were included. Exclusion criteria included case reports, single-patient series, in vitro studies, and abstracts without full publication. This methodology was designed to balance breadth and clinical relevance, highlighting evidence most pertinent to clinicians while following SANRA principles for high-quality narrative reviews.

Guideline foundations and core principles

Major international guidelines recommend a patient-focused, multidisciplinary approach with exercise, weight reduction, and education of the patient as the basis for management.

Osteoarthritis Research Society International (OARSI)

Emphasizes a general "core set" of non-pharmacologic management in all patients that includes formal exercise (aerobic, strengthening, neuromuscular, aquatic, or mind-body), weight reduction in obese or overweight patients, and continuing education. Interventions should be based on patient preference, need, and comorbidities, and tailored where feasible. The use of assistive devices such as bracing and walkers is recommended only in select cases. Pharmacologic therapy is considered adjunctive; oral and topical NSAIDs are initial management for relief of symptoms, with intra-articular corticosteroids considered an adjunct to therapy for a short period if prior therapy fails to relieve patient symptoms [4,6].

American College of Rheumatology (ACR) and Arthritis Foundation (AF)

Supports daily, prolonged exercise of any kind, weight loss in overweight or obese patients, self-efficacy or self-management interventions, and patient education. Supervised behavior assistance and intervention are reported to be advantageous by the guideline; Tai Chi, cane use, tibiofemoral bracing, and topical NSAIDs are also endorsed. Oral NSAIDs and intra-articular corticosteroids are likewise indicated for symptom management. Acetaminophen, duloxetine, and tramadol are conditionally endorsed for modest efficacy or safety concerns [1,7].

European League Against Rheumatism (EULAR)

Consistent with the rest of the societies in supporting education, exercise, and weight reduction. Bracing and walking aids are to be utilized as necessary. Pharmacologic therapy, primarily topical and oral NSAIDs, is reserved for chronic pain [4].

National Institute for Health and Care Excellence (NICE)

Recommends exercise as the initial treatment with subsequent weight loss if obese/overweight and self-management/education. Walking sticks and bracing are recommended as needed. Pharmacological treatment, including topical and oral NSAIDs, is recommended only after failure of non-pharmacologic intervention. Finally, intra-articular corticosteroid injections may be used for short-lasting relapses [1].

European Society for Clinical and Economic Aspects of Osteoporosis, Osteoarthritis, and Musculoskeletal Diseases (ESCEO)

Also recommends exercise, weight loss, and education as first-line treatment within a multidisciplinary approach. Pharmacologic therapy is adjunctive with a preference for topical or oral NSAIDs and short courses of oral corticosteroids [6].

Non-pharmacologic interventions such as exercise, weight loss, and patient education are the cornerstone of management for all presented guidelines. Pharmacologic and procedural treatments are adjuncts for refractory symptoms, with therapy being individualized and based on shared decision-making. The guideline recommendations are summarized in Table 1 [1-3,6,7].

**Table 1:** Summary of recommendations from major international guidelines for nonoperative knee osteoarthritis management OARSI: Osteoarthritis Research Society International; ACR: American College of Rheumatology; AF: Arthritis Foundation; EULAR: European League Against Rheumatism; NICE: National Institute for Health and Care Excellence; ESCEO: European Society for Clinical and Economic Aspects of Osteoporosis, Osteoarthritis, and Musculoskeletal Diseases; NSAIDs: Non-steroidal antiinflammatory drugs.

Intervention	OARSI	ACR/AF	EULAR	NICE	ESCEO
Education & self-management	Core	Strong	Core	Core	Core
Exercise	Core	Strong	Core	First-line	First-line
Weight loss	Core	Strong	Core	First-line	First-line
Bracing / cane	Select cases	Strong	Recommended	As needed	As needed
Topical NSAIDs	Recommended	Strong	Not addressed	Prefer over oral	Preferred
Oral NSAIDs	Recommended	Recommended	Not addressed	After non-pharm	Preferred pharmacologic
Intra-articular corticosteroid	Conditional	Strong	Not addressed	For short-term flares	Short course
Hyaluronic acid	Against	Conditional against	Not addressed	Do not offer	Selected patients
Platelet-rich plasma	Strongly against	Strongly against	Not addressed	Do not offer	Not addressed

Non-pharmacological interventions

Patient Education & Self-Management

Patient education and self-management are central components of nonoperative care for knee OA, enhancing adherence, coping ability, and informed decision-making. All major international guidelines identify education initiatives as core management strategies. Education provides patients with a clear understanding of OA etiology, prognosis, and treatment options, fostering realistic expectations and enabling active participation in care decisions.

Self-management programs, often multidisciplinary and group-based, combine skill development such as goal setting or problem-solving, guidance on medication and joint protection, and strategies to maintain physical activity. These programs reliably improve self-efficacy, encourage sustained engagement in exercise and weight control, and reduce pain and functional impairment, even when effect sizes are modest. The referenced guidelines strongly endorse self-efficacy and self-management programs for knee OA based on consistent benefit and minimal risk [7].

Education and self-management also address pain-related fear, stress, and depression while providing tools for self-monitoring and modifying patient behavior. These approaches support long-term adherence to exercise and lifestyle modifications, which are essential for symptom control and preserving function [3,6,7]. Incorporating them into standard clinical practice is widely recommended for achieving optimal outcomes [6,7]. 

Exercise & Physical Activity

Strength training, aerobic exercise, and balance program interventions enhance pathophysiological and functional impairments of primary knee OA and lead to less pain and improved function. As discussed, they are recommended as first-line therapies by the presented international guidelines [1-3,7-9].

Strength training aids periarticular and quadriceps muscle function, joint stability, lowers mechanical load, and alleviates pain. Quadriceps-specific exercise, particularly supervised a minimum of three times per week, is noted as optimal. Moderate intensity aerobic exercise such as walking or cycling improves cardiovascular fitness, preserves weight, and may lower systemic inflammation. Balance and neuromuscular training addresses proprioceptive deficits and functional instability, thereby lowering the risk of falls and improving confidence [3,4,7,8-11].

Beyond guideline recommendations, broader studies indicate strength training, quadriceps-specific exercises, and aerobic exercise have modest benefits on function and pain, without one being superior than another. Thus, recommendations should be patient-specific and based on preference and resources [7-10]. The literature supports that patient‑specific, supervised exercise programs improve compliance and clinical outcomes, with benefits that persist for several months after the formal program concludes [2-4,7-9].

Weight Management

High-quality evidence from randomized controlled trials and meta-analyses establishes that weight loss among obese or overweight individuals with knee OA decreases joint load, relieves pain, and enhances function. Biomechanically, one pound of weight loss is equivalent to decreasing roughly four pounds of compressive load transmitted through the knee during activities of daily living [12].

Clinically, weight loss equivalent to five percent or greater of baseline weight significantly enhances function and pain, and further improvement is noted beyond a 10 percent reduction in weight [7,12]. The literature supports that interventions with diet in addition to exercise show optimal outcomes with weight loss equivalent to seven percent of baseline or greater [13].

Successful weight control interventions are the result of pairing diet counseling and behavioral interventions with exercise, as multicomponent interventions that can include group and individual counseling, follow-up, and social cognitive theory for maximal compliance [12,13]. Education and self-management components allow for skill development and problem-solving to create long-term changes in lifestyle [7,12].

Biomechanical Aids

Orthotics, canes, and braces are beneficial adjuncts in select patients. Patients with reduced stability may benefit from assistive devices such as canes or tibiofemoral bracing, when device‑tolerant and experiencing significant symptomatic instability. Other types of bracing such as patellofemoral devices have mixed support in the literature and international guidelines given their heterogeneity of benefit and patient comfort [7].

For medial compartment OA, valgus or “unloader” braces reduce medial joint load and create short-term functional and pain reduction but are likely to be hindered by pain [14,15]. For these patients, contralateral canes have also been shown to reduce load, enhance stability, and reduce pain [7]. Orthotics such as laterally-wedged insoles have shown variable benefit and are not as widely recommended [14,16].

Physical Modalities

Unlike other musculoskeletal conditions, the literature highlights the limited role of interventions such as transcutaneous electrical nerve stimulation (TENS) and therapeutic ultrasound in the management of knee OA. TENS is related to short-term pain relief but has a less consistent and less remarkable effect on function compared to exercise, bracing, or weight loss [17,18].

Therapeutic ultrasound was shown to yield small-to-moderate functional and pain benefits over placebo, particularly at higher intensities, over 24 treatments in a four to eight week period. Although the data is limited, therapeutic ultrasound and TENS can be used as adjunct treatments, but should not replace first-line recommendations such as exercise and weight loss [19-21].

Mind-Body & Complementary Therapies

Mind-body approaches such as Tai Chi, yoga, and acupuncture can provide moderate relief in pain, function, mobility, and psychological status. Tai Chi has the most evidence, with most randomized trials showing significant pain reduction and decrease in stiffness, balance, and quality of life. The outcomes are reported as effective as those of standard physical therapy [22,23].

Mind-body practices, like Tai Chi or Qigong and yoga also have positive effects, but the evidence is less comprehensive [24,25]. Both improve depression and mental health more than that seen with resistance or aerobic exercise [26].

Acupuncture also has potential benefits in pain and disability, but variable quality evidence has provided tentative suggestions. Mind‑body treatments are safe and potentially useful adjuncts, particularly for patients seeking holistic benefits or concomitant improvement in mood [22,23,26,27].

Pharmacologic treatment

NSAIDs & Analgesics

Oral and topical NSAIDs are strongly recommended as the first-line pharmacologic therapy. Some guidelines recommend topical NSAIDs over oral formulations given their equal effectiveness and lower gastrointestinal, cardiovascular, and renal risk [2,7]. Oral NSAIDs are still appropriate for more widespread or more severe symptoms but at lowest dose and for shorter duration [7,28].

However, many guidelines are in agreement that NSAIDs need to be supplemented by fundamental non-pharmacologic treatments, and augmented by biomechanical devices, physical modalities, and mind-body interventions [1,7].

Intra-articular Injections

Corticosteroids: Cause immediate relief of pain, within four to six weeks and for up to three months. Multiple injections (>three to four per year) indicate virulent cartilage loss and thus are reserved for short-term relief of an acute flare [2,29,30].

Hyaluronic acid (HA): Produces small-to-moderate functional/pain improvements with peak effect at three to six months. Longer duration of relief can be achieved with higher molecular weight preparations, and it provides the greatest benefit for mild-to-moderate OA and for patients with NSAID intolerance [29-31].

Platelet-rich plasma (PRP): The evidence for PRP is polarized. Some studies provide evidence for efficacy in pain relief and function that compares to HA and corticosteroids. PRP may be considered primarily for younger patients with mild‑to‑moderate osteoarthritis; leukocyte‑poor PRP formulations are generally better tolerated than leukocyte‑rich preparations [30-33]. However, some guidelines like AAOS have no recommendations for its routine use for knee OA.

Adjunctive Agents

Serotonin-norepinephrine reuptake inhibitors (SNRIs) like duloxetine can also be recommended in the event of ongoing pain despite optimal non-pharmacologic therapy and NSAIDs, or in the presence of coexisting depression or widespread pain syndromes. Duloxetine provides modest improvements in pain and function for some patients, though its use may be limited by adverse effects such as nausea and sedation [1,7,34].

Opioid agonists like tramadol are conditionally utilized for intractable pain when NSAIDs and other therapy are ineffective or contraindicated, though their use should be limited by virtue of diminished benefit and potential for dependence [1,3,7,34]. Acetaminophen is of poor analgesic efficacy and carries the risk of hepatotoxicity. It is used intermittently in NSAID-intolerant patients. Topical capsaicin is also recommended but in isolated circumstances [34-36].

Not Recommended

Botulinum toxin, fish oil, glucosamine, chondroitin, and disease-modifying agents without proven benefit are not recommended given their lack of supportive evidence [7,37,38]. Other disease-modifying agents like hydroxychloroquine, methotrexate, bisphosphonates, and biologics have shown to provide no improvement [7,39].

A practical summary of treatment alternatives, along with their expected benefits, typical candidates, and key cautions, is provided in Table 2, and is intended to complement the narrative above and to facilitate quick clinical reference. [2,7,28-36,40-51]. 

**Table 2:** Pharmacologic and injection options for knee osteoarthritis: typical benefit, candidates, and major cautions NSAIDs: Non-steroidal antiinflammatory drugs; GI: Gastrointestinal; CV: Cardiovascular; CNS: Central nervous system; RCT: Randomized controlled trials.

Intervention	Typical benefit & durability	Optimal candidates/when to consider	Major cautions
Topical NSAIDs	Analgesia while used; best for localized knee pain	First-line analgesic option, especially in older adults or those with GI/CV risks	Local skin reactions; avoid over large/broken skin
Oral NSAIDs	Short-term analgesia and function gains while on therapy	Flares or persistent pain unresponsive to topical agents	GI bleed/ulcer, kidney injury, CV risk—use lowest effective dose, shortest duration
Acetaminophen	Mild analgesia, often modest/temporary	Rescue or when NSAIDs contraindicated	Hepatotoxicity at high doses or with alcohol
Duloxetine	Pain reduction in some patients within weeks; benefit persists while on therapy	Co-existing widespread pain and/or depression	Nausea, somnolence; serotonin syndrome risk with other serotonergic drugs
Tramadol (weak opioid)	Short-term analgesia; use sparingly	Last-line if other options fail/contraindicated	Dizziness, dependence, GI/CNS adverse effects
Intra-articular corticosteroid	Short-term relief ~2–10 weeks (peaks early)	Acute flares, significant synovitis; bridge to rehab	Transient hyperglycemia; potential cartilage effects with frequent/repeated use
Hyaluronic acid	Onset weeks; benefit often seen ≥12 weeks; can last ~3–6 months	Patients preferring to avoid/limit NSAIDs or after corticosteroids; shared decision-making	Post-injection flare; cost; variable products
Platelet-rich plasma	Several RCT/meta-analyses show pain/function gains that may persist 6–12 months in some protocols	Select patients willing to pay, aiming to delay surgery; heterogeneity requires informed consent	Product heterogeneity; cost; coverage gaps
Genicular nerve radiofrequency ablation	Pain relief commonly 3–6 months, sometimes up to 6–12 months	Refractory pain, poor surgical candidates or deferring arthroplasty; after failure of core care/injections	Procedure risks (neuritis, numbness); technique-dependent outcomes
Genicular artery embolization	Early studies/pooled analyses show symptom improvement; durability 12–24 months in some cohorts	Refractory moderate–severe pain in specialized centers; when surgery is contraindicated or deferred	Access/site pain; non-target embolization (rare); limited long-term RCTs

Emerging and innovative therapies

Genicular Artery Embolization (GAE)

GAE is a vigorous treatment, which, according to reports, has produced great pain relief and quality of life improvement in mild-to-moderate OA that has not responded to conservative care. Current literature reports studies with 78-92% of patients experiencing ≥50% relief of typical pain at six to 12 months [40-43]. Side effects for GAE are usually classified as harmless and self-limiting [41,42,44]. GAE remains outside of standard OA practice guidelines but is endorsed by the Society of Interventional Radiology in properly selected candidates who are not eligible for operation [45,46].

Genicular Nerve Radiofrequency Ablation (RFA)

RFA suppresses pain transmission by destroying the genicular nerve branches. Recent studies from the literature have shown greater short-term relief of pain and function compared with corticosteroids, HA, NSAIDs, acetaminophen, and placebos [15,47-51]. Improvement is documented as greatest at six months, when 74% of patients experienced ≥50% pain relief, which can persist for as long as 12 months in some patients [48,49].

Intravascular Laser Irradiation of Blood (ILIB)

ILIB is not established in OA guidelines and has poor-quality evidence. Early evidence elsewhere provides promising anti-inflammatory effects, but evidence is lacking for OA [52].

Gene Therapy

Experimental, disease‑modifying approaches targeting inflammatory pathways (e.g., IL‑1β inhibition) and anabolic pathways (e.g., intra‑articular tumor growth factor (TGF)‑β1 or fibroblast growth factor (FGF)‑18 delivery) have shown promising results in preclinical studies and early‑phase clinical trials. Transgene expression over many years alleviated inflammation and cartilage loss in animal models [53-56]. However, regulatory approval of any OA gene therapy has not been clearly established.

Bone Marrow Aspirate Concentrate

Bone marrow aspirate concentrate (BMAC) is derived from bone marrow harvested through aspiration, then processed to yield a concentrate of mesenchymal stem cells and cytokines which is then administered via an intra-articular injection. Some studies report significant short- to mid-term pain and functional value, but without perceived superiority to adipose-derived stem cell therapy (ADSC) or other orthobiologics such as PRP [57-60]. In the literature, both BMAC and adipose-derived stromal vascular fraction (SVF) have shown symptomatic benefit, with SVF offering superior pain relief, but functional outcome was equal for both modalities [58,61-63]. Long-term outcomes demonstrate sustained benefit up to four years in some cohorts, but cumulative evidence is inconsistent due to heterogeneity of preparation and patient recruitment [59,64].

Adipose-Derived Stem Cells (ADSC)

ADSCs are harvested from fat tissue, usually through liposuction, and concentrated for use as an injectable orthobiologic. Some studies have showed that the infrapatellar fat pad (IFP) can be harvested arthroscopically as a viable and consistent source of ADSCs. In these studies, cells attached and grew in culture, had clonogenic potential, and exhibited multilineage differentiation, including robust chondrogenic capacity. Notably, IFP-derived stromal vascular fraction cells had greater CD105 expression than the other adipose sources, consistent with greater regenerative potential [65].

Umbilical Cord Stem Cells

Umbilical cord-derived mesenchymal stem cells (UC-MSCs) are isolated from postnatal umbilical tissue and processed as an injectable biologic with potential anti-inflammatory and regenerative effects. Some studies have shown moderate improvement in function and pain at short to intermediate term follow-up, with a safety profile equivalent to BMAC and ADSC therapy, with no concerning adverse events reported in the available literature [66-70]. The available comparison studies reveal that both UC-MSCs and ADSC provide symptomatic relief, with the greatest improvement in pain for the ADSCs and the best functional result for the UC-MSCs on the Western Ontario and McMaster Universities Osteoarthritis Index (WOMAC) measure [61,71].

Hematopoietic Stem Cells

Hematopoietic stem cells (HSC) do not play a role in the management of knee OA due to the focus on mesenchymal stem cell (MSC) therapies, such as BMAC, adipose tissue, or UC-MSC [66-72]. HSC, the progenitors of blood lineages, have limited research for their applications in OA management and thus are not recommended for intra-articular injection in knee OA.

Corticosteroid formulations

Methylprednisolone acetate, triamcinolone acetonide, and betamethasone all induce short-term pain relief when administered as intra-articular corticosteroid injections for knee O, with maximal effect at one to six weeks and fading at 12-24 weeks. Their side effect profiles are similarly comparable, with most being self-limiting local reactions like pain, swelling, and infrequently infection or post-injection flare. Clinical comparative trials prove that methylprednisolone acetate has possibly more intense analgesic effect up to six weeks than triamcinolone acetonide and betamethasone but there is no difference in their effect by 12 weeks, with comparable functional benefit or duration of action after that point [73-78]. Betamethasone and triamcinolone acetonide are equivalent to methylprednisolone acetate in terms of overall pain and function outcomes, although triamcinolone hexacetonide has shown more intense and immediate pain relief in some series [74-78]. Given that no differences in systemic adverse effects or cartilage toxicity have been observed among the different corticosteroids in the setting of single or infrequent doses, major guidelines have not established a preference between corticosteroid alternatives for intra-articular injection.

Integrated multidisciplinary care and implementation barriers

Optimal care is achieved through collaboration among physical therapists, physicians, dietitians, and allied health professionals. This leads to concerted, tailored, and evidence-based care. Obstacles to optimal OA care include disparities in weight-management resources, variable availability of supervised exercise, and lack of compliance with long-term activity regimens [79].

## Conclusions

Nonoperative care remains the foundation of knee OA management. Lifestyle adaptation, structured education, formal exercise programs, and weight loss are consistently recommended as first-line treatments, with pharmacotherapy, biomechanical aids, and selected intra-articular injections serving as adjuncts when symptoms persist. At the same time, newer therapeutic alternatives are generating growing interest. While early results are promising, evidence is still limited by the heterogeneity of protocols, short follow-up, and modest sample sizes. 

Future research priorities include high-quality comparative efficacy studies of emerging interventions against established care; extended follow-up to clarify the durability of benefits; phenotype-directed treatment strategies; and scalable models of multidisciplinary care to improve access, adherence, and real-world implementation. Patient-targeted, team-based approaches remain the most promising pathway to sustainable improvements in pain, function, and quality of life for individuals living with knee OA.
